# Trends and Immediate Outcomes of Syncope During Pregnancy: A Narrative Review

**DOI:** 10.7759/cureus.49833

**Published:** 2023-12-02

**Authors:** Rafael Cortorreal Javier, Parth Vikram Singh, Jeena Shrestha, Rasha Abdalla, Punay Narang, Harshkumar Patel, Kameshwar P Yadav, Tirath Patel, Olatunji E Fadiora, Humayun Shahzad, Kiran Abbas

**Affiliations:** 1 Department of Medicine, Universidad Iberoamericana (UNIBE), Santo Domingo, DOM; 2 Department of Medicine, Indira Gandhi Government Medical College, Nagpur, IND; 3 Department of Medicine, Jalalabad Ragib Rabeya Medical College, Sylhet, BGD; 4 Department of Medicine, Shendi University, Shendi, SDN; 5 Department of Medicine, Spartan Health Sciences University, Vieux Fort, LCA; 6 Department of Medicine, Pandit Dindayal Upadhyay (PDU) Medical College, Rajkot, IND; 7 Department of Medicine, Universal College of Medical Sciences, Bhairahawa, NPL; 8 Department of Medicine, American University of Antigua, St. John's, ATG; 9 Department of Medicine, Windsor University School of Medicine, Cayon, KNA; 10 Department of Medicine, Punjab Medical College, Faisalabad, PAK; 11 Department of Community Health Sciences, Aga Khan University, Karachi, PAK

**Keywords:** treatment, pregnancy, maternal health, incidence, fetal health, diagnosis, cardiac syncope

## Abstract

Pregnancy-related syncope presents special difficulties due to the rapid physiological changes that occur throughout gestation. This narrative review provides a thorough summary of the patterns and pregnancy outcomes secondary to syncope during pregnancy. There is an increase in syncope burden during pregnancy, and hence it is critical that medical professionals understand the significance of syncope during pregnancy. Syncope can have a negative impact on the health of the mother as well as the fetus. Therefore, this review summarizes data from studies on syncope in pregnancy. It includes observational studies, case reports, and review articles. Early detection and proper management are very important because pregnant women who experience cardiac syncope are at risk of unfavorable neonatal and maternal outcomes. The review reveals diverse trends in syncope incidence during pregnancy, emphasizing the need for a nuanced understanding of temporal variations. Risks of injury, uteroplacental insufficiency, psychological effects, interruptions in prenatal treatment, possible aggravation of pre-existing diseases, and lifestyle changes are examples of immediate maternal repercussions. Pregnancy-related syncope is a complex condition that affects the health of the mother and the fetus. The study stresses the need for careful clinical treatment due to the rapid results and the diversity in incidence patterns. The unique component of a possible relationship to the brain health of offspring justifies further investigation in this area.

## Introduction and background

Syncope, characterized as a transient, abrupt loss of consciousness with an accompanying loss of postural tone from which recovery is spontaneous, represents a significant clinical concern when manifesting in pregnant women [1]. The prevalence of syncope in pregnancy is not fully elucidated; however, it is known to be a common occurrence, especially due to physiological changes that predispose expectant mothers to episodes of reduced cerebral blood flow [1]. Some studies report that dizziness and syncope presenting late in pregnancy can account for about 10% of cases [2].

During pregnancy, hemodynamic shifts, including increases in blood volume and cardiac output, along with hormonal effects on the vasculature, can contribute to a heightened risk of syncope. This risk is particularly pronounced given that syncope typically presents in a bimodal age distribution, notably affecting individuals between 10 and 30 years old, a range that encompasses many pregnant women [1].

Syncope and recurrent presyncope are common among pregnant women. The associated symptoms may be very troubling, and these women may receive poor advice and little reassurance from their obstetric care providers [3-5]. The rationale for this review is underscored by the potential consequences syncope bears during pregnancy. While often a benign vasovagal phenomenon, syncope in expectant mothers can occasionally herald more serious underlying conditions, such as cardiac arrhythmias [3], and pose risks of maternal injury from falls. Furthermore, it may signal cardiovascular instability that could jeopardize fetal well-being, making its assessment critical in prenatal care [4]. The clinical significance of syncope in pregnancy is amplified by the limited data available regarding its impact on maternal and fetal outcomes [1], and by the intricate balance clinicians must maintain between maternal and fetal health, especially when considering interventions for arrhythmia-associated syncope [6].

Pregnancy-induced changes in the cardiovascular system are known to significantly correlate with syncope, which itself is a significant marker for cardiovascular disease [7]. The timing of syncopal episodes in pregnancy carries potential implications, signaling potentially adverse pregnancy outcomes [8-10]. As such, understanding the trends of syncope in pregnant women, from incidence and diagnosis to treatment, is crucial for maternal and fetal health and well-being.

The objective of this study is to consolidate and distill the available literature concerning the trends and immediate outcomes of syncope in pregnant women. By providing a comprehensive synthesis of current knowledge, this review aims to enhance the understanding of syncope in this unique population, thereby improving diagnostic and management strategies to ensure the safety and well-being of both the mother and fetus.

## Review

Methodology

A comprehensive search strategy was employed to identify relevant literature about syncope during pregnancy. Different electronic databases, including PubMed, ScienceDirect, Scopus, and Google Scholar, were explored. To make our search strategy comprehensive, we ensured the inclusion of a combination of Medical Subject Headings (MeSH) and keywords relevant to "syncope" and "pregnancy." Boolean operators (AND, OR) were also utilized to form combinations. The present study included observational studies, case reports, and review articles. Studies involving pregnant women experiencing syncope episodes, irrespective of the underlying cause, were eligible for inclusion. Only articles published in English were included. Studies involving non-pregnant populations, pediatric patients, or unrelated conditions were excluded from the review. Additionally, studies lacking detailed information on the syncope episodes, outcomes, or interventions were excluded to maintain the quality and relevance of the included literature.

Epidemiology of syncope in pregnancy

The prevalence of syncope during pregnancy varies across studies, with reported rates ranging from 0.3% to 9.0% of pregnancies. A study conducted by Gibson et al. found that 4.6% of pregnant women experienced syncope during pregnancy [11]. Another prospective study by Moolla et al. reported a higher prevalence among pregnant women with hypertrophic cardiomyopathy (HCM), with syncope occurring in 9.0% of pregnancies, with increased frequency in the first trimester [12]. These variations in prevalence rates can be attributed to differences in study populations, diagnostic criteria, and methodologies used in different studies. A recent study by Chatur et al. revealed that the overall incidence rate for syncope in pregnancy is 9.7 per 1000 pregnancies (95% CI, 9.4-10.0 per 1000 pregnancies) in Alberta, Canada [10]. They also revealed that the incidence of syncope among pregnant women increased from 2005 to 2014, thus raising concerns about this phenomenon. This increase may reflect a growing awareness and better reporting of syncope events or potential changes in population health and pregnancy-related factors.

Similarly, a retrospective cohort study was conducted including all singleton deliveries occurring between the years 1991 and 2021 at a large tertiary medical center. The study population included 232,475 pregnancies, 774 (0.3%) of which were affected by maternal syncope, which most frequently first occurred during the second trimester (44.5%), followed by the first trimester (31.8%) and finally the third trimester (27.7%) [13]. There is an increase in the incidence of syncope in pregnancy and this warrants further exploration of the phenomenon.

Trends and patterns

There is limited information on the patterns of syncope during pregnancy, although certain studies have indicated that there is a higher chance of syncope during the first trimester. This increased risk is probably caused by vasovagal processes, hormonal variations, and hemodynamic alterations [13,14]. Furthermore, data indicates that women who already have cardiovascular disease or a history of syncope may be more susceptible to syncopal episodes during pregnancy [10,13]. The physiological changes during pregnancy, including increased blood volume, decreased vascular resistance, and hormonal fluctuations, can lead to altered autonomic regulation, making pregnant women more susceptible to syncope [15,16]. Moreover, the vena cava may be compressed by the growing uterus, lowering cardiac output and venous return. This can lead to syncope, especially when the patient is in a supine position [17,18].

Pathophysiology of syncope in pregnancy

Vasovagal syncope, orthostatic hypotension, neurological reasons, and cardiac arrhythmias are the most frequent causes of syncope [16]. Some are more serious than others, but all are always a matter of concern since they can have a negative impact on maternal or neonatal outcomes, as will be discussed in later sections. It is also challenging to perform a thorough work-up for a pregnant patient with syncope, since there is a risk of exposing the fetus to unnecessary radiation. Based on the literature, the following have been identified as the main causes of syncope.

Hemodynamic Changes

Blood volume and cardiac output both significantly increase during pregnancy to satisfy the needs of the growing fetus. Nonetheless, when the woman stands upright, this extra blood volume may cause vasodilation and reduced vascular resistance, which might result in orthostatic hypotension and syncope [19,20].

Hormonal Influences

Variations in hormone levels, especially elevated progesterone, can impact the tone of vascular smooth muscle and cause vasodilation. Hormonal fluctuations can also affect the sensitivity of the baroreceptor reflex, which is responsible for controlling blood pressure. These hormonal changes might be factors in syncope and orthostatic intolerance [4,15].

Vasovagal Mechanisms

During pregnancy, vasovagal syncope is frequently brought on by emotional or orthostatic stimuli. Syncope can occur when emotional stress triggers the vasovagal reaction, causing a rapid decrease in blood pressure and heart rate [21,22]. This reaction may be exacerbated by the expanding uterus's pressure on the vena cava, particularly when the individual is in a supine position. This lowers cardiac output and venous return [17,18].

Cardiac Factors

Pregnant women may experience arrhythmias, such as supraventricular tachycardia, which can cause syncope [8,13]. Structural heart diseases or congenital heart conditions that were previously asymptomatic can become symptomatic during pregnancy due to the increased cardiac workload, potentially leading to episodes of syncope [23-25].

Orthostatic Hypotension

Pregnant women frequently experience orthostatic hypotension due to hormonal changes and increased blood volume [26,27]. Sudden changes in posture, particularly when rising from a supine or sitting position, can result in a rapid drop in blood pressure, which may trigger syncope [28]. This risk is exacerbated in women with pre-existing conditions affecting blood pressure regulation [25,27].

Dehydration and Anemia

Dehydration and anemia are common during pregnancy and can exacerbate the risk of syncope. Insufficient fluid intake and low hemoglobin levels reduce blood volume and oxygen-carrying capacity, respectively, which can compromise cardiovascular stability [29]. Adequate hydration and monitoring of hemoglobin levels are crucial in preventing syncope related to these factors.

Maternal and fetal outcomes of syncope

Only limited studies have explored the maternal and neonatal outcomes of syncopal events during pregnancy. Furthermore, some evidence reveals contrasting findings, such as that in a study by Muppa et al., which revealed no significant difference in the incidence of pregnancy complications in patients with vasovagal syncope compared to healthy cohorts [21]. A similar conclusion was offered by Rodriguez-Manero et al., revealing that syncope during pregnancy was not correlated with worse pregnancy outcomes [30]. However, evidence dictates that syncopal episodes in pregnancy must never be taken lightly as they are associated with maternal and neonatal complications. Chatur et al., revealed that the rate of preterm birth was higher in pregnancies with syncope during the first trimester (18.3%) compared to those without syncope (15.0%; P<0.01) [10]. Those with preexisting cardiac issues, such as hypertrophic cardiomyopathy (HCM), are at increased risk for morbidity and mortality. A study by Moolla et al., revealed that pregnant women with HCM had a death incidence of 0.2%. Moreover, syncopal events were experienced by 9% of them. The study concluded that HCM in pregnancy was associated with outcomes including postpartum hemorrhage, neonatal death, stillbirth, and preterm [12]. This highlights the criticality of prompt intervention in cases of syncope episodes in pregnancy. According to Orenshtein et al., there are a number of unfavorable consequences linked to maternal syncope, or fainting, during pregnancy. In particular, regardless of when the syncope occurs during pregnancy, there is a 52% increased risk of intrauterine growth restriction (IUGR), especially with syncope in the first trimester, a 33% higher chance of cesarean delivery, and a 79% increased risk of long-term neurological morbidity in offspring. These findings demonstrate the substantial effect that maternal syncope has on the mother's and the child's short- and long-term health outcomes [13].

Yao et al.'s case study of a pregnant lady, age 31, shows how syncope may be extremely harmful to expectant mothers [31]. At 29 weeks of pregnancy, the lady developed increasing palpitations that were recurrent and related to syncope. These repeated syncopal episodes had a severe and incapacitating effect on her, preventing her from returning to her regular activities and job. In this instance, syncope was linked to a decrease in systolic blood pressure; nonetheless, spontaneous recovery occurred.

Table 1 highlights the existing literature on syncope in pregnancy and the main outcomes associated with it.

**Table 1:** Syncope in pregnancy and its outcomes

Author	Study Design	Study Aims	Study Population	Primary Outcome	Main Findings
Deveau et al. [22]	Secondary data analysis	To investigate the variations in vasovagal syncope recurrence, provoking variables, and clinical presentation between men and women	Patients in Prevention of Syncope Trials (POST) I and II	Differences in clinical presentation, outcomes, and time to first syncope between men and women	9.0% of pregnant women had syncope in the study. Additionally, 91.7% of pregnant women who had vulvar varicosities (VVs) had heat as a provoking element, compared to 8.3% of non-pregnant women (p=0.049).
Chatur et al. [10]	Retrospective cohort study	To assess the incidence of syncope during pregnancy, examine temporal trends, and evaluate outcomes	Pregnant women	Incidence of syncope during pregnancy	Temporal trends showed variations in syncope incidence, and outcomes were diverse, requiring further investigation.
Schwarzwald et al. [16]	Case report	Presented a case of a 28-year-old pregnant woman who presented with suspected cardiac syncope	Pregnant woman	Diagnosis and management of recurrent syncope in pregnancy	The case report focuses on a pregnant patient with a history of recurrent syncope who, while having normal cardiac imaging results, showed a substantial decrease in blood pressure on a tilt table test and sinus tachycardia on an implanted loop recorder.
Orenstein et al. [13]	Retrospective cohort study	To explore the incidence of syncope during pregnancy, immediate pregnancy outcomes, and offspring's long-term neurological health	Pregnant women	Incidence of syncope, immediate pregnancy outcomes, offspring neurological health	Syncope during pregnancy associated with adverse immediate outcomes, and potential impact on offspring neurological health was suggested, requiring further research.
Yarusi et al. [9]	Case report	To present a rapidly evolving case of syncope during pregnancy	Pregnant woman	Clinical presentation, diagnostic evaluations, management, and outcomes of a syncope case during pregnancy	Described a complex case of syncope during pregnancy, emphasizing the challenges in diagnosis and management.
Yarlagadda et al. [8]	Case report	Presenting a case of 22-year-old primigravid	Pregnant woman	A 26-week woman presented with dizziness, light-headedness, nausea, diaphoresis, and syncope	The patient regained consciousness and her vitals were within normal range after the episode. In conclusion, syncope during pregnancy is a common incident however, it is mostly benign and self-limited.
Huang et al. [28]	Case report	At 34 weeks of pregnancy, a 23-year-old lady experienced recurrent syncope	Pregnant woman	Imaging findings	Imaging revealed that the patient suffered syncope every time she shifted from a supine to a sitting posture due to a significant collapse of the inferior vena cava.
Yao et al. [31]	Case report	Presenting a 29 weeks-pregnancy, a 31-year-old working woman with recurring, progressive palpitations associated with syncope	Pregnant woman	Case management	Multiple syncopal events were recorded, highly debilitating as she was unable to resume her work and normal day activities. There was an associated drop in systolic blood pressure with spontaneous recovery. She was prescribed metoprolol with immediate resolution of symptoms and episodes. She delivered a healthy baby at term.
Kamata et al. [32]	Case report	Presenting a case of a 32-year-old woman with sudden-onset syncope in the second trimester	Pregnant woman	N/A	An unusual case when a pregnant lady, without labor or obstetric assistance, experienced abrupt syncope and disseminated intravascular coagulation at 14 weeks. This occurrence was most likely caused by an early second-trimester amniotic fluid embolism, and subchorionic hematoma was suspected.

Discussion

This section provides an opportunity to summarize our findings and discuss the gaps in the literature and future direction of research. Pregnancy-related syncope presents special difficulties because of the intricate interactions between physiological changes, possible causes, and different outcomes. The complex character of syncope in pregnant women is highlighted in this review, underscoring the need to comprehend the underlying pathophysiological processes. It is clear that a variety of causes, including hemodynamic alterations, hormonal effects, vasovagal processes, cardiac issues, and autonomic nervous system imbalances, can lead to syncope. These risks are further compounded by the physiological adjustments associated with pregnancy [10-13]. It should be noted that pregnant women with no pre-existing conditions can also develop syncope. One such case was reported by Ciorsti MacIntyre, where a pregnant woman with no prior history of structural cardiac disease presented with syncope [33]. The patient presented with arrhythmia and syncopal episodes. The study emphasized the increasing trend of atrial fibrillation, which is becoming more prevalent in pregnancy and may present with syncope. This requires immediate intervention as there is an associated risk of cardiac morbidity and mortality for the woman and the fetus [33].

Our data highlights the significance of customized techniques for diagnosis and treatment. Healthcare professionals need to be vigilant for pregnant patients who are more vulnerable, such as those with a history of syncope or pre-existing cardiovascular issues. Improved maternal and fetal outcomes can be achieved through proper monitoring measures, lifestyle improvements, and medical care when needed.

When treating syncope in pregnant women, clinicians need to employ a comprehensive and customized strategy. Obtaining a thorough medical history is of utmost importance, with a special focus on syncope episodes and any risk factors. Frequent blood pressure and heart rate monitoring can help identify hemodynamic abnormalities early, particularly in high-risk pregnancies. Pregnant individuals can lower their risk of syncopal episodes by modifying their lifestyle and learning about potential triggers such as abrupt postural changes and mental stress. In order to reduce the risk of syncope, clinicians should prioritize managing underlying cardiovascular problems and establishing optimum control [34,35]. Comprehensive treatment can also be facilitated by a multidisciplinary approach, combining cardiologists, obstetricians, and other experts. Professionals should stress the significance of routine prenatal care, including continuous follow-ups, to ensure a safe and healthy pregnancy for both mother and fetus. Despite advancements in our understanding of syncope in pregnancy, many questions remain unanswered. Further research is needed to determine how syncope affects fetal outcomes, especially long-term outcomes.

## Conclusions

In summary, syncope during pregnancy poses a complicated and diverse set of challenges for patients and medical professionals. This narrative review has clarified the numerous facets of syncope during pregnancy, from its epidemiology and causes to its potential effects on maternal and fetal outcomes. The review of the literature highlights the necessity for a comprehensive understanding of this phenomenon and emphasizes the need for individualized approaches to diagnosis, treatment, and prevention. Despite recent advancements, there remain many unanswered questions, which call for further study and collaboration among experts, researchers, and clinicians. Prospective studies, incorporating diverse pregnant populations and long-term follow-ups, are crucial to unraveling the intricacies of syncope during pregnancy.
